# Neuron to Oligodendrocyte Precursor Cell Synapses: Protagonists in Oligodendrocyte Development and Myelination, and Targets for Therapeutics

**DOI:** 10.3389/fnins.2021.779125

**Published:** 2022-01-18

**Authors:** Daniela M. S. Moura, Emma J. Brennan, Robert Brock, Laura A. Cocas

**Affiliations:** ^1^Department of Biology, Santa Clara University, Santa Clara, CA, United States; ^2^Department of Neurology, University of California, San Francisco, San Francisco, CA, United States

**Keywords:** synapse, myelin, ion channel, gaba receptor, glutamate receptor, oligodendrocyte precursor cell (OPC), multiple sclerosis

## Abstract

The development of neuronal circuitry required for cognition, complex motor behaviors, and sensory integration requires myelination. The role of glial cells such as astrocytes and microglia in shaping synapses and circuits have been covered in other reviews in this journal and elsewhere. This review summarizes the role of another glial cell type, oligodendrocytes, in shaping synapse formation, neuronal circuit development, and myelination in both normal development and in demyelinating disease. Oligodendrocytes ensheath and insulate neuronal axons with myelin, and this facilitates fast conduction of electrical nerve impulses via saltatory conduction. Oligodendrocytes also proliferate during postnatal development, and defects in their maturation have been linked to abnormal myelination. Myelination also regulates the timing of activity in neural circuits and is important for maintaining the health of axons and providing nutritional support. Recent studies have shown that dysfunction in oligodendrocyte development and in myelination can contribute to defects in neuronal synapse formation and circuit development. We discuss glutamatergic and GABAergic receptors and voltage gated ion channel expression and function in oligodendrocyte development and myelination. We explain the role of excitatory and inhibitory neurotransmission on oligodendrocyte proliferation, migration, differentiation, and myelination. We then focus on how our understanding of the synaptic connectivity between neurons and OPCs can inform future therapeutics in demyelinating disease, and discuss gaps in the literature that would inform new therapies for remyelination.

## Introduction

Myelination is critical for signal conduction within axons, and demyelination is an essential feature of traumatic brain injury and stroke, as well as degenerative diseases such as Alzheimer’s disease and multiple sclerosis. Twenty years ago, Bergles and Jahr (2000) discovered the presence of functional synaptic contacts between neurons and NG2+ oligodendrocyte precursor cells (hereafter referred to as OPCs). They found evidence of both excitatory (Bergles and Jahr, 2000) and inhibitory (Lin and Bergles, 2004a) synaptic input onto OPCs, and found that these synapses were present throughout the brain in gray matter in the hippocampus (Bergles et al., 2000; Mangin et al., 2008), cerebellum (Lin et al., 2005) and the cochlear nucleus (Müller et al., 2009), and in white matter (Kukley et al., 2007; Etxeberria et al., 2010). OPCs are the only glial cell type that receives neuronal synapses, although mature OLs, astrocytes, and microglia all express unique complements of neurotransmitter receptors, synaptic adhesion proteins, and gap junctions that are important for homeostasis, metabolism, and in the case of OLs, myelin formation; this is reviewed elsewhere (Pocock and Kettenmann, 2007; Allen and Eroglu, 2017; Butt et al., 2019). Synaptic activity between neurons and OPCs is known to influence OPC proliferation and differentiation into mature oligodendrocytes (OLs) (Reviewed by Bergles et al., 2010) and is important for myelination and remyelination, discussed in detail here. Because these processes are critical for proper myelination, understanding ion channel expression and function in OPC development and maturation as well as their role in synaptic transmission can inform future therapeutic interventions for demyelinating diseases and brain injuries where myelin damage has occurred.

In this review, we discuss recent developments in research on neuron to OPC synapse function, including the expression and function of key neurotransmitter receptors in OPCs and the roles of voltage-gated channels in these synapses. We review recent findings on the effects of glutamatergic and GABAergic synapses between neurons and OPCs on myelination. We address how neuron to OPC synapses can promote proliferation versus differentiation, the mechanisms behind the loss of functional synapses during development, and the developmental changes in receptor expression to update the current OPC review literature. Finally, we posit that the literature on channel function in OPCs can inform our understanding of the etiology of MS and other demyelinating disease, and propose possible new therapeutic directions.

Twenty years after Bergles and colleagues first discovered neuron to OPC synapses, they remain enigmatic: we know much about neuron to OPC synaptic properties and their direct impact on OPC depolarization, as well as the specific channel expression in OPCs, and their role in OPC development and maturation, reviewed here. Gaps remain, however: we know little about downstream signal transduction as a result of this synaptic activity, or how this is important for plasticity; we discuss this as well. Finally, we have not yet harnessed this knowledge to leverage new therapeutics for remyelination, and we conclude the review with some possible future directions for therapies after demyelination.

## Glutamatergic Receptor Expression, Development, and Function in Oligodendrocyte Precursor Cells

Early in development, OPCs are relatively homogenous in their receptor expression, electrophysiological properties, gene and protein expression, but as they mature, they become a heterogeneous population with differential gene expression, with only a subpopulation of cells differentiating into myelinating OLs, de la Fuente et al. (2020) reviewed in Bergles and Richardson (2015). Previous work has shown that OPCs receive glutamatergic input from neurons. Early work from the Bergles lab found that these currents were α-Amino-3-hydroxy-5-methyl-4-isoxazolepropionic acid (AMPA-type) receptor mediated, and present in both the developing and mature brain (Bergles and Jahr, 2000). Further, the properties of these synapses have all of the essential features of neuron to neuron synapses: fast activation, quantal responses, presynaptic inhibition, and both facilitation and depression (Bergles and Jahr, 2000). While the presence of these synapses early in OPC development suggests that they may be important for developmental myelination, myelin is remodeled throughout life, and it is possible that neuron to OPC synapses are instructive for both initial myelination and for myelin remodeling later in life.

### Amino-3-Hydroxy-5-Methyl-4-Isoxazolepropionic Acid Receptor Expression Changes and Importance for Oligodendrocyte Precursor Cell Proliferation, OL Differentiation, and Myelination

**FIGURE 1:**
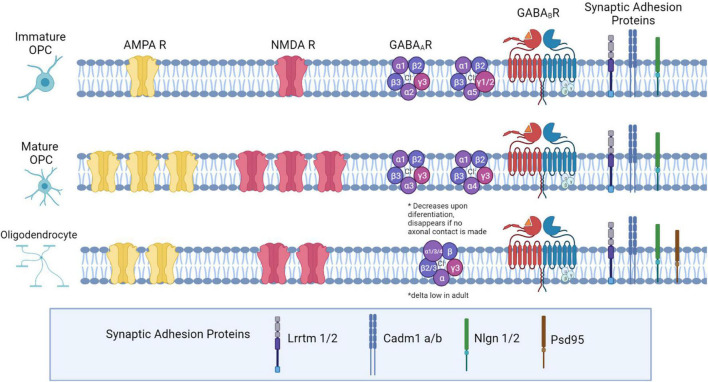
Neurotransmitter receptor expression and synaptic adhesion protein expression across the Oligodendrocyte lineage. In immature OPCs (PO-P9), AMPA and NMDA receptors are present at low densities, with expression peaking later in development (P10-P35). As OPCs mature into OLs, they express fewer AMPA and NMDARs. GABAA receptor subunit expression changes: in early development, α1,α2, and α5 subunits are highly expressed. Later in development, α2 and α5 decrease expression, and α3 and α4 both increase in expression. β2 and β3 expression is high, and does not change during maturation, while β1 declines from low to very low expression in OPCs later in development. The δ subunit is not present in development but is expressed in a small percentage of OPCs in adulthood. All γ subunits are expressed in OPCs: γ3 is highly expressed and maintained throughout development, but γ1 and γ2 are highly expressed in OPCs early in development and decrease in adulthood. Both GABA_*B*_ subunits are expressed consistently throughout development (GABA_*B1*_ and GABA_*B2*_). Both OPCs and mature OLs express the adhesion proteins Lrrtm1, Lrrtm2, Neuroligin 1, Neuroligin 2, Cadm1a, and Cadm1b, based on RNASeq expression analysis in OPCs, and based on LOF function analyses in OLs. Additionally, OLs express PSD-95.

The surface expression of the glutamatergic AMPA receptor changes through the OL lineage: AMPA receptor expression and activation by glutamate begin very early in development, and the receptor density increases as OPCs mature (Figure 1; Spitzer et al., 2019). Kainate-mediated KA/AMPA currents are detected as early as P0, and peak at P20-35, but both current and receptor density remain high through 9 months of age. Further, channel expression in gray matter vs. white matter is developmentally regulated: before P9, KA/AMPAR channels are similarly expressed in gray and white matter; after P9, channels density is higher in the cortex and lower in the corpus callosum. However, upon OPC differentiation into mature oligodendrocytes, there is a roughly 12-fold decrease in surface expression density of AMPA receptors. This change also corresponds to a decrease in mRNA expression for all AMPA subunits (De Biase et al., 2010). These data may be explained by a decrease in the number of OPCs and an increase in the number of OLs later in life.

**FIGURE 2:**
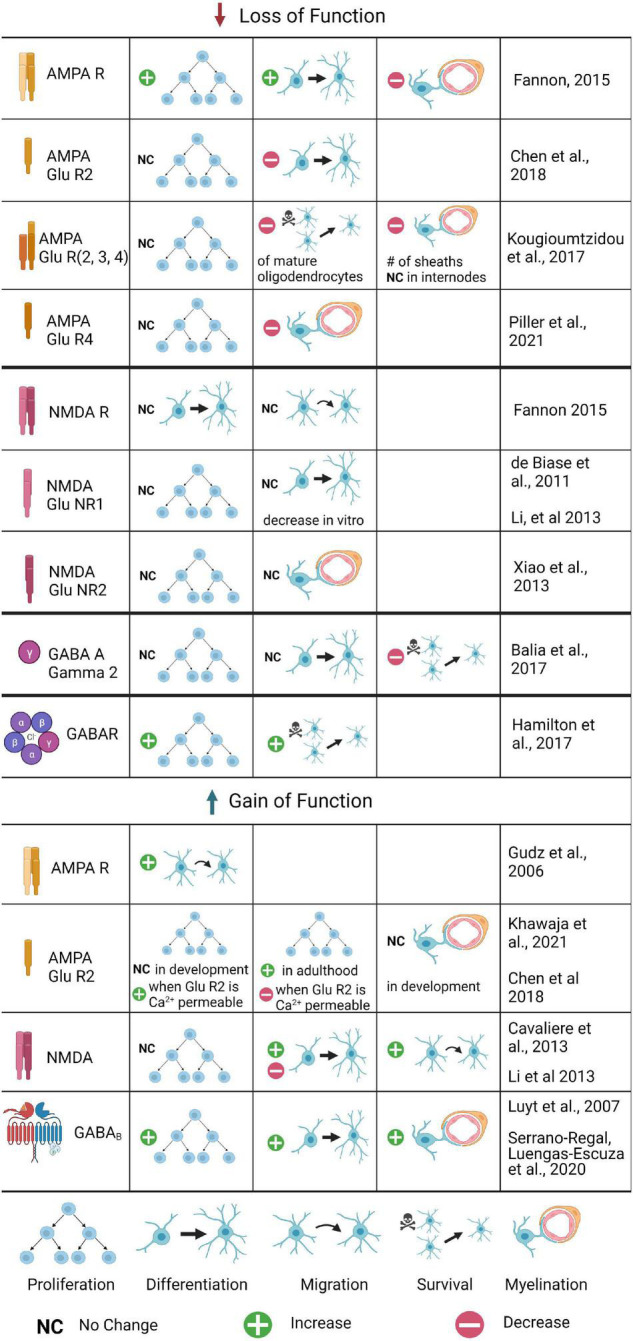
The role of glutamatergic and GABAergic receptors in OPC development and myelination: Summary of Gain and Loss of Function Experiments. Changes in OPC proliferation, differentiation, migration, or survival are included, as well as changes in myelination. Loss of function experiments have been conducted for several AMPA and NMDA receptor subunits, as well as the GABAA γ2 subunit; gain of function experiments are limited to AMPA, AMPA GluR2, NMDA, and GABAB experiments. Where the process was tested but no differences found, no change is noted; if the process was untested it is not included for that receptor. Where conflicting results were found, both are listed.

AMPA receptors are tetrameric; the Ca^2+^ permeability of AMPA depends on the presence of specific subunits in the tetramer (Reviewed by Wright and Vissel, 2012). The GluA2 subunit is transcribed containing the amino acid glutamine (Q) but modified post-translationally to replace the glutamine with arginine (R) in most receptors. This edited subunit, GluA2(R), renders AMPA impermeable to Ca^2+^. Only AMPARs without GluA2 or containing GluA2(Q) are Ca^2+^ permeable. This has critical implications for the role of AMPA in neuron to OPC synapses. It is not known whether the expression of GluA2 changes during OPC development, however, we have clues about its purpose from gain of function experiments. OPCs that overexpress GluA2 in development do not show changes in proliferation or differentiation, but overexpression of GluA2 in OPCs in adulthood leads to increased OPC proliferation (Figure 2; Khawaja et al., 2021). Since the addition of GluA2 renders AMPA receptors Ca2+ impermeable, this suggests that decreasing Ca2+ in OPCs in development does not affect OPC proliferation, but that AMPA mediated Ca2+ signaling is important for OPC proliferation in the mature brain. Other findings are consistent with this conclusion: another group created a triple knockout of GluA2/3/4, which effectively abolishes all AMPA mediated inputs to OPCs, and found that this loss of AMPA-mediated synaptic input led to decreased survival of mature OLs and a 20% decrease in the number of myelin sheaths, but no decrease in OPC proliferation, indicating that OL survival but not OPC proliferation is dependent upon AMPA mediated neuronal inputs (Kougioumtzidou et al., 2017). However, several other recent studies provide contradictory but equally compelling findings, making it clear that the role of AMPA-signaling in OPC development and myelination remains unresolved. Another group compared unedited (Ca2+ permeable) AMPAR to AMPARs that had been edited with pore-dead GluA2 subunits, and found that both conditions increased proliferation of OPCs and decreased differentiation into OLs (Chen et al., 2018). This finding indicates that Ca2+ entry through AMPARs is important for OPC proliferation, and that Ca2+ inhibits differentiation of OPCs into OLs.

Oligodendrocyte precursor cells and immature OLs also express GluA4, which also renders AMPA receptors calcium permeable. Recent work using a GluA4 mutant and *in vivo* time lapse imaging in zebrafish has shown that OPC migration (but not proliferation) is disrupted: OPCs migrate more slowly and travel shorter distances in the spinal cord (Piller et al., 2021). In the GluR4 mutants, myelination was also disrupted, as measured by a decrease in myelin internode length. Activation of voltage gated Ca2+ channels rescued both migration and myelination defects, demonstrating that Ca2+ entry is mediating both of these defects and suggesting that it is normally important both for OPC migration and myelination. How do we resolve these conflicting findings about the role of AMPA-mediated Ca2+ in OPC proliferation, differentiation, and myelination? There are differences in the timing and the magnitude of the loss of function in these studies (a constitutive knock out of GluA2/3/4 subunits in the Kougioumtzidou et al. (2017) paper vs. viral mediated disruption of Ca2+ signaling in GluA2 mutants in the Chen et al., 2018 paper, for example): perhaps loss of all receptors early in development results in compensatory mechanisms that are not possible later in development. The downstream signaling pathways that promote OPC survival in development may be different than those that maintain the OPC pool in adulthood.

Previous work has shown that OPCs express AMPARs, that glutamate leads to OL depolarization and Ca2+ rise in OLs and in OPCs via either via axonal stimulation or exogenous glutamate application, and that OPCs have an intermediate Ca2+ permeability and variable receptor composition (Seifert and Steinhäuser, 1995; Bergles et al., 2000). More recently this effect has shown to be mediated by both AMPARs and P/Q and L-type voltage gated Ca2+ channels (Barron and Kim, 2019). In glutamate uncaging studies, OPCs produce an inward current that can be confirmed by blocking AMPA using the antagonist CNQX (De Biase et al., 2010; Kukley et al., 2010). However, this current halves as OPCs mature into pre-OLs, and finally in OLs, inward current is not blocked by application of AMPA antagonist, indicating the downregulation of AMPA expression and the upregulation of other glutamatergic receptors (Kukley et al., 2010). This suggests that AMPA receptor mediated signaling may be important in OPCS to induce differentiation into OLs, as well as to serve as a signal from electrically active neurons to pre-myelinating oligodendrocytes to begin myelinating active axons. The purpose of these small, likely kainate-mediated inward currents in mature OLs is less clear, but could be useful for directing targeting of myelin onto axons, or regulating myelin node length on axons.

### NMDA Receptor and Subunits in OPC Development

Another key ionotropic receptor mediating glutamatergic synapses is N-methyl-D-aspartate, or NMDA (Reviewed by Paoletti et al., 2013). NMDA ion channel function is distinct from that of AMPA because all NMDARs are highly Ca^2+^ permeable and require a change in membrane voltage to remove a Mg2+ block prior to channel activation. NMDA receptor expression increases until OPCs differentiate and begin to express myelinating genes, when it peaks and then declines in OLs (Figure 1). In addition, NMDA also decreases much sooner in OPCs located in gray matter regions of the brain but persists in white matter regions (Spitzer et al., 2019). NMDAR currents are developmentally regulated, peaking at P6-P16 and declining until NMDA current is very low at 3 months of age; NMDAR density also declines rapidly to zero (De Biase et al., 2011; Spitzer et al., 2019). This is consistent with the maturation of OPCs and the decrease in their potential to make myelin, and corresponds to a loss of synaptic contacts in maturing OPCs. The types of subunits expressed do not change between OPCs and OLs (all three, GluN1, GluN2A, and GluN2B, are, at least early in postnatal development) (Li et al., 2013). Two GluNR1 subunits are required for a functional receptor, thus this subunit can be knocked out to study the lack of NMDA-mediated transmission (De Biase et al., 2011).

The precise role and importance of NMDA in neuron to OPC synapses is debated. Some studies show NMDA has no active role in OPC properties or maturation: GluNR1 knockout OPCs show no significant changes in resting membrane properties or number of EPSCs (De Biase et al., 2011). Furthermore, researchers observed no difference in OPC proliferation, morphology, or OL differentiation (De Biase et al., 2011; Fannon et al., 2015; Figure 2). Because of this, several researchers have concluded that NMDA mediated currents are not critical for OPC development; instead, the presence of NMDA in these synapses is only to support the function of AMPA. For example, NMDA knockouts exhibit a 27% increase in Ca^2+^ permeable AMPA (De Biase et al., 2011). NMDA could provide negative feedback to control the Ca^2+^ permeability of AMPA and its corresponding effects on differentiation. Uniquely, NMDARs in OLs have a weaker Mg2+ block than that found in neurons, and indeed, AMPA and NMDA receptors are spatially segregated in OLs (Káradóttir et al., 2008). However, other studies have contested these claims and shown NMDA is critical for neuron to OPC synapses. These studies conclude that indeed NMDA activity does not affect OPC proliferation, but promotes OPC migration, which was blocked by NMDA antagonists and knockouts of the critical NMDA subunits *in vitro* (Li et al., 2013). NMDA activity also increased the differentiation in OPC cultures, followed by an increase in myelination and complexity of OPCs, resulting in longer processes and increased branching *in vitro* (Li et al., 2013). These effects were blocked in NMDA antagonists or RNAi for NMDARs *in vitro*, and were also abolished when Tiam1, a Rac1-GEF, was pharmacologically inhibited or knocked down *in vitro*, indicating that NMDAR signaling activates the Tiam1/Rac1/ERK signaling cascade, at least *in vitro*. The conflicting results *in vitro* and *in vivo* do not negate the interesting results of the signaling cascade data, which now need to be confirmed *in vivo* with conditional knockouts or RNAi, and point to the possibility of compensatory signaling via AMPARs, which also conduct Ca2+ and could potentially activate the ERK signaling pathway when AMPAR receptors are absent.

### Glutamatergic Synapses

Glutamate is known to be released onto OPCs via neuronal synapses, resulting in small excitatory postsynaptic potentials; however, propagating action potentials have not been observed in OPCs (De Biase et al., 2010; Osterstock et al., 2018). Glutamatergic synapses between neurons and OPCs have been demonstrated by induction of glutamate vesicle release from presynaptic neurons, either through chemical or electrical stimulus of surrounding axons and paired recordings of OPCs (Bergles and Jahr, 2000; Lin and Bergles, 2002; Jabs et al., 2005; Ziskin et al., 2007; De Biase et al., 2010). Release of presynaptic glutamate vesicles induces inward excitatory postsynaptic currents (EPSCs) in OPCs while other receptors and channels are blocked (De Biase et al., 2010; Kukley et al., 2010). Miniature EPSCs (mEPSCs), measured in the presence of TTX to isolate the effects of glutamate release from other effects of action potentials in presynaptic neurons, also have similar amplitudes and kinetics throughout OPCs in the brain, indicating consistent glutamate receptor densities (De Biase et al., 2010). Electrical train stimulation of axons to release glutamate also causes EPSCs in OPCs; EPSCs occur both during the train and after (Nagy et al., 2017). Large unitary EPSC amplitude distribution indicates release of multiple glutamate vesicles onto OPCs (Nagy et al., 2017). OPCs also react differently in response to changes in frequency (Hz) and intensity of stimulus within the presynaptic neuron. Repetitive axon stimulation increases glutamate release to OPCs, which in turn facilitates higher amplitudes of EPSCs (Nagy et al., 2017). These effects are increased by stimulation at higher frequencies or trains of stimulation at lower frequencies. Repetitive stimulation also results in desynchronization of glutamate release as calcium ions build up in the presynaptic neuron, causing increased delayed EPSCs after stimulation ceases. The rate of delayed EPSCs is higher with longer stimulus trains and higher frequencies (Nagy et al., 2017). This could provide a mechanism for adaptation and plasticity, addressed later. Finally, recent work has used monosynaptic viral circuit tracing to confirm that neuron to OPC synapses are present throughout the brain, and are robust: whisker trimming did not change these connections in the somatosensory cortex (Mount et al., 2019).

These variations in OPC responses suggest OPCs can discriminate between types of glutamatergic transmission; therefore, neurons may use glutamatergic synapses to direct differentiation and myelination. Differences in stimulation appear to direct either differentiation or proliferation. *In vivo* stimulation generally increases the number of pre-OLs and decreases the number of OPCs, although lower frequencies increase differentiating cells more effectively and higher frequencies increase proliferation (Nagy et al., 2017). In addition, OPCs demonstrate synchronous responses to glutamate, meaning EPSCs are detected immediately after extracellular stimulation. However, glutamatergic signaling produces an asynchronous response in pre-OLs, with increased latency and decreased amplitude (Kukley et al., 2010). This discrepancy can be accounted for by downregulation of AMPA, but the magnitude of the change indicates there is also a difference in glutamate release from the presynaptic neuron. The delayed EPSCs in pre-OLs likely result from the dismantling of vesicle-release mechanisms in the pre-synaptic neurons. Because of this, glutamatergic signaling, or lack thereof, could contribute to OPC differentiation. mEPSCs continue to decline overall in pre-OLs and OLs (De Biase et al., 2010). In addition to presynaptic signaling mechanisms, the changes in postsynaptic expression of glutamatergic receptors and voltage-gated channels could affect how glutamatergic synapses direct OPC maturation (Spitzer et al., 2019).

The effect of OPC depolarization on neuronal synaptic function has been recently addressed. Researchers used an optogenetic approach to activate OPCs in the alveus of the hippocampus, then measured changes in conduction velocity of axons using CAPS recordings in the subiculum of the hippocampus (Ge et al., 2006). They found that OPC depolarization resulted in faster conduction velocities in these axons, resulting in increased excitatory synaptic responses in bursting (but not regular firing) pyramidal neurons. Changes in neuronal plasticity were also induced: optogenetically increasing OPC depolarization during theta burst stimulation lead to an increase in LTP (long term potentiation) as measured by EPSC amplitude, whereas optogenetic inhibition during theta burst stimulation led to a decrease in LTP, as measured by EPSC amplitude (Ge et al., 2006). LTP in OPCs is mediated via AMPARs, rather than NMDARs, as is the case in neuronal LTP: using AMPA antagonists but not NMDA antagonists abolished LTP induction in OPCs. Importantly, this effect was mediated by Ca2+ permeable, Glur2-lacking AMPARs in OPCs, suggesting that the downstream consequences of LTP induction in OPCs (increased Ca2+/CAMKII signaling, immediate early gene activation, induction of plasticity proteins, and growth factors) would be similar in OPCs. However, to date, the significance of this plasticity in OPCs has not been examined further, leaving an open question of the role of LTP in OPC function. We know that loss of function experiments that block neuronal NMDA-mediated LTP lead to decrease in OPC proliferation as well as OPC neurite outgrowth, suggesting that neuronal plasticity might be important for OPC maturation (Zhang et al., 2018).

## GABAergic Receptor Expression, Development, and Function in Oligodendrocyte Precursor Cells

### GABA_A_ Receptor and Subunits in Oligodendrocyte Precursor Cell Development

γ-aminobutyric acid (GABA) receptors are the primary receptors for inhibitory synaptic transmission in the brain. GABA_*A*_ is an ionotropic receptor permeable to chloride and bicarbonate ions. Work from Bergles and colleagues have found that activating GABAergic interneurons results in transient and rapid inward Cl-currents in OPCs (Lin and Bergles, 2004b; Tanaka et al., 2009). However, GABA has a depolarizing effect as OPCs, like immature neurons, have a very high concentration of intracellular chloride ions, and do not express the KCl co-transporter KCC2, resulting in anions flowing out of OPCs in response to GABA_*A*_ receptor activation, causing small depolarizations in OPCs (Lin and Bergles, 2004b; Tanaka et al., 2009); reviewed in Serrano-Regal et al. (2020b). This effect of GABA on OPCs may be different between gray and white matter: there is immunohistochemical and pharmacological evidence that KCC2 is expressed in OLs in the optic nerve, although whether it is expressed in OPCs or other white matter areas is not known (Malek et al., 2003). OPCs are the only class of glial cells known to express GABA_*A*_, as astrocytes and NG2-negative glial cells fail to respond to GABA_*A*_ agonists (Labrada-Moncada et al., 2020). GABAergic currents in OPCs are also blocked by GABA_*A*_ antagonists (Zonouzi et al., 2015).

The GABA_*A*_ receptor has five subunit components that can be any combination of the 19 possible different subunits. The subunit composition of GABA_*A*_ receptors is varied in OPCs and changes throughout development (Figure 1). Much like glutamate receptors, there is a switch from synaptic to extra synaptic localization of GABA_*A*_ receptors as OPCs differentiate (Balia et al., 2017). In early development, α1,α2, and α5 subunits are most abundantly expressed, and a smaller percentage express α3; later in development, α2 and α5 both decline in expression, and α3 and α4 both increase in expression (Passlick et al., 2013). β2 and β3 are abundant: they are present in over 50% of OPCs, and their expression does not change during maturation, while β1 declines from low to very low expression in OPCs later in development. The δ subunit is not present in early development but is expressed in a small percentage of OPCs in adulthood. Finally, all γ subunits are expressed in OPCs: γ3 is the most widely expressed, and is maintained at high levels throughout development, while γ1 and γ2 are highly expressed in OPCs early in development but decline in adulthood (Passlick et al., 2013; Balia et al., 2015). Changes in GABAR subunits are consistent with a switch from synaptic to extrasynaptic signaling later in development, which we will discuss later in this review. For example, the application of an α5 inverse agonist reduced current in OPCs early in development but not later in development (Balia et al., 2017). Similarly, the application of zolpidem and Zn2+ increased GABA-evoked responses in OPCs at synapses, and the authors showed through a series of experiments that these effects were likely mediated by synaptic γ2 expression, and that extrasynaptic GABARs were unlikely to express γ2 (Passlick et al., 2013).

The γ2 subunit has been shown to have a key role in OPC development as well as GABAergic synapses between neurons and OPCs. Knocking out γ2 from GABA_*A*_ in OPCs decreases the frequency of PSPs (Figure 2; Balia et al., 2017). The presence of γ2 also affects the types of synapses formed between OPCs and neurons. Fast spiking interneurons have been shown to target OPCs at sites containing GABA_*A*_ receptors with the γ2 subunit. Non-fast spiking interneurons are more likely to form synapses at sites that do not contain γ2 (Orduz et al., 2015). These γ2-mediated GABAergic synapses do not affect proliferation and differentiation in OPCs. However, they are known to affect the density and survival of OPCs during development. Typically, OPC numbers peak in early postnatal development and decline near the end of development, at approximately P30 in mice (although OPCs are present throughout life in low numbers). When γ2 is knocked out, OPC density continues to increase at this time, but there is no increase in differentiation (Balia et al., 2017). These data indicate that γ2 is required to regulate the density of OPCs.

### GABA_B_ Receptor and Subunits in Oligodendrocyte Precursor Cells

GABA_*B*_ receptors are G-protein coupled metabotropic receptors. When activated by GABA, GABA_*B*_ receptors reduce cyclic AMP, inactivate voltage-gated calcium channels, and activate inward-rectifying potassium channels (reviewed in Gaiarsa and Porcher, 2013). Unlike GABA_*A*_ receptors, GABA_*B*_ receptors are only composed of two subunits: GABA_*B1*_ and GABA_*B2*_. Also, while GABA_*A*_ subunit expression changes over time and during differentiation, both GABA_*B*_ subunits are expressed consistently throughout development (Figure 1). GABA_*B*_ function has not been widely studied in OPCs, and emerging research has not yet clarified their importance in neuron to OPC synapses. Investigators found that OPCs express both GABA_*B1*_ and GABA_*B2*_ subunits (Luyt et al., 2007), and expression was higher in OPCs than in OLs (Serrano-Regal et al., 2020b). There are conflicting results regarding the role of GABAB in OPCs. One recent study showed the application of a GABA_*B*_ agonist does not trigger any response in OPCs (Labrada-Moncada et al., 2020). However, other studies found different effects *in vitro*: GABA_*B*_ activation led to increased OPC proliferation (Luyt et al., 2007) and application of a GABA_*B*_ agonist increased differentiation and myelination *in vitro*, while a GABA_*A*_ antagonist had no effect on accelerating differentiation (Serrano-Regal et al., 2020b). OPCs cultured with dorsal root ganglia neurons (DRG) in the presence of GABA differentiate and myelinate DRG axons. This effect is mediated by GABA_*B*_ receptors but not GABA_*A*_ receptors, as these cells no longer express GABA_*A*_ receptors, indicating that downstream signaling via activation of second messenger cascades via GABA_*B*_ activation is important for GABA-induced myelin formation (Serrano-Regal et al., 2020b). More research is needed to further understand the role of GABA_*B*_ receptors in OPC development, and their role at neuron to OPC synapses.

As OPCs mature, their GABA receptor expression changes, and this differential expression is dependent upon myelination state. OPCs that mature into OLs but do not contact axons lose GABA_*A*_ expression entirely, while those OLs that contact axons and myelinate them decrease their GABA_*A*_ expression but maintain GABA_*B*_ expression as they mature into OLs, at least in DRG-OPC co-cultures (Arellano et al., 2016). This maintenance of GABA_*B*_ expression is also present in forebrain and optic tract OPCs (Serrano-Regal et al., 2020b). The downstream effects of the activation of GABA_*B*_ signaling are not well understood, although evidence supports CREB mediated myelin gene activation through a number of possible pathways, including MAPK, PI3K, and FAK (reviewed in Serrano-Regal et al., 2020b). Understanding how these downstream pathways regulate myelin gene activation may help promote remyelination after injury. The role of GABA_*B*_ in OPCs as they mature into OLs suggests that they may be important in regulating myelin sheath length and thickness, although this is not well understood.

### GABAergic Synapses

Oligodendrocyte precursor cells also receive GABAergic synapses from inhibitory neurons and has been shown to lead to depolarization through the efflux of Cl^–^ ions (Kirchhoff and Kettenmann, 1992; Lin and Bergles, 2004a; Jabs et al., 2005; Tanaka et al., 2009). There is some controversy over whether this depolarization also effects the intracellular Ca2+ concentration of OPCs: Slice physiology data indicates that depolarization in OPCs is insufficient to open VGCCs (Lin and Bergles, 2004a). However, calcium imaging experiments in dissociated OPCs indicate changes in intracellular Ca2+ concentrations in OPCs but not in mature OLs (Kirchhoff and Kettenmann, 1992; Tanaka et al., 2009; Arellano et al., 2016). This may be due to different conditions in culture vs. intact brain slices where the interneuron to OPC synapse is intact; regardless, the role of depolarization in OPC function is still poorly understood. GABAergic transmission varies by OPC age, indicating a possible role for GABAergic synapses in regulating OPC development: spontaneous GABA-induced currents can be observed in OPCs as soon as postnatal day three, but the frequency of these currents declines sharply in the second postnatal week (Vélez-Fort et al., 2010; Serrano-Regal et al., 2020b). As a result, direct GABAergic transmission may be most significant early in OPC development. Though the frequency of GABA-induced currents declines through development, currents can still be induced in later postnatal weeks. However, these currents have different properties than those during early development. Later currents have slower rise and decay, but their amplitudes do not change. This suggests that these currents are being caused by GABA spillover from synapses being detected by receptors outside the synaptic cleft, as it would take longer for GABA to diffuse to and activate these receptors (Vélez-Fort et al., 2010). Combined with the change in receptor subunit composition, these factors suggest a switch to extrasynaptic GABA communication in later development (Vélez-Fort et al., 2010; Balia et al., 2015; Serrano-Regal et al., 2020b).

A major role for GABAergic synapses is the regulation of OPC survival and myelination. Application of GABA antagonists dramatically reduces the number of OPCs undergoing apoptosis and increases OPC proliferation (Hamilton et al., 2017). OPC proliferation peaks and then many OPCs undergo apoptosis in control mice between P13 and 30, but GABA receptor γ2 knock-out mice have an increase in the OPC pool, suggesting that GABA signaling is important for OPC self renewal (Figure 2; Balia et al., 2017). Further, application of GABA increases OPC process branching, number of myelin segments, and MBP expression in DRG-OPC co-cultures in the presence of GABA_*A*_ inhibitors, suggesting that these effects are mediated through GABA_*B*_ second messenger signals (Serrano-Regal et al., 2020b). GABA_*B*_ signaling also promotes myelination in experiments done in the rat sciatic nerve in early developmental stages (Corell et al., 2015). In addition, increased OPC proliferation has also been noted when GABAergic activity is reduced due to neonatal hypoxia (Zonouzi et al., 2015), although this is a pathological condition that affects many pathways, and other factors are likely to contribute to this change in OPC proliferation. GABA regulation of OPC proliferation and myelination are not simply due to inhibition of neuronal activity: blockade of action potentials does not create similar effects on proliferation and apoptosis (Hamilton et al., 2017). This indicates that GABA release may exert a direct effect on OPCs at synapses, particularly through GABA_*B*_ mediated signaling, but active neurons trigger separate mechanisms that also affect OPC proliferation and apoptosis. Indeed, OPCs cultured alone in the presence of GABA do not proliferate (Serrano-Regal et al., 2020a). Further, increasing GABAergic activity onto OPCs via optogenetics or genetically inactivating interneuron synapses onto OPCs does not impair or enhance the proliferation or differentiation of OPCs; nor does it impair the global amount of myelin, or the number of myelinated nodes in the cortex, suggesting that GABAergic signaling is either dispensable for OPC development and myelination, or that homeostatic compensation is possible when all GABAergic signaling is lost (Balia et al., 2017; Ortolani et al., 2018). Indeed, recent work has shown an important role for GABAergic activity specifically from PV (parvalbumin) expressing interneurons in myelination in the cortex (Benamer et al., 2020). Myelination of PV axons is required for feed-forward inhibition in cortical circuits, and disrupting GABAergic signaling in OPCs via deletion of the GABAA receptor γ2 subunit prior to myelination resulted in disruption of myelin distribution on PV axons, with abnormal myelin at branch points and longer nodes and internodes. These myelination defects reduced the normally high firing frequency of PV neurons, and decreased their connectivity, resulting in an excitation-inhibition imbalance (Benamer et al., 2020). Additional work detangling the downstream signaling pathways of GABAergic and glutamatergic signaling onto OPCs will be key to understanding the role of neuronal circuits in OPC function and myelination in the future.

## Voltage-Gated Channels and Their Role in Oligodendrocyte Precursor Cell Development and Maturation

### Roles of Ca2+ and VGCCs in Oligodendrocyte Precursor Cell Depolarization, Proliferation, and Myelination

**FIGURE 3:**
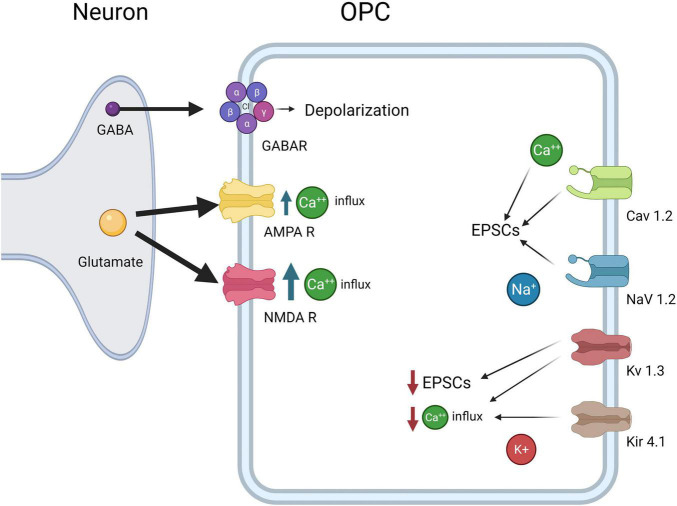
OPC electrophysiological Properties. OPCs express CaV1.2, which results in influx in Ca2+ and increases EPSCs, NaV1.2, which results in influx in Na+ and increases in EPSCs, Kv1.3 which decreases EPSCs and Ca2+ influx, and Kir4.1, which decreases Ca2+ influx. Both glutamate receptors AMPAR and NMDA lead to increased Ca2+ concentrations inside OPCs, although NMDARs allow more Ca2+ influx than AMPARs. Finally, GABAA causes depolarization of the OPCs as the intracellular Cl- concentration is high in OPCs.

Intracellular calcium concentrations are key regulators of OPC development and myelination. As in neurons, voltage-gated channels are responsible for the depolarization of OPCs. EPSCs in OPCs are caused by Ca^2+^ transients, and Ca^2+^ flux is modulated by the voltage-gated Na^+^ and K^+^ channels (Figure 3). Voltage-gated channels for calcium, potassium, and sodium have been studied and mRNA for several types of voltage-gated Ca^2+^ channels (VGCCs) has been found in OPCs. Transcripts for L-type channels Cav1.2 and 1.3 and T-type Cav3.1 and 3.2 are most common, though transcripts for the P/Q and N-type channels Cav2.1 and 2.2 have also been found to a lesser extent in OPCs (Haberlandt et al., 2011; Cheli et al., 2015). VGCCs are essential for depolarization in OPCs, and Cav1.2 is the major player in Ca^2+^ influx and EPSCs. Multiple labs have shown that Cav1.2 knockouts lead to the greatest decrease in Ca^2+^ transients in OPCs (Cheli et al., 2015, 2016; González et al., 2017). In control cells, OPCs show a marked increase in intracellular Ca^2+^ in response to depolarization while Cav1.2 knockout OPCs show less than 50% of baseline levels (Cheli et al., 2016). VGCCs also contribute to the spreading of depolarization throughout the dendritic spines. The Ca^2+^ signal recorded in OPCs rises steeply over time and does not diminish in amplitude after initial stimulation, indicating that VGCCs on other branches are being opened (Sun et al., 2016). Some studies have suggested Ca^2+^ influx in OPCs is mediated primarily by sodium-calcium exchangers (NCX) rather than VGCCs (Tong et al., 2009), but more recent research suggests that NCXs are not involved in EPSCs in OPCs (Sun et al., 2016; Labrada-Moncada et al., 2020). Ca2+ signaling is highly implicated in OPC proliferation, although the downstream signaling pathways have not been extensively explored (Paez and Lyons, 2020).

**FIGURE 4:**
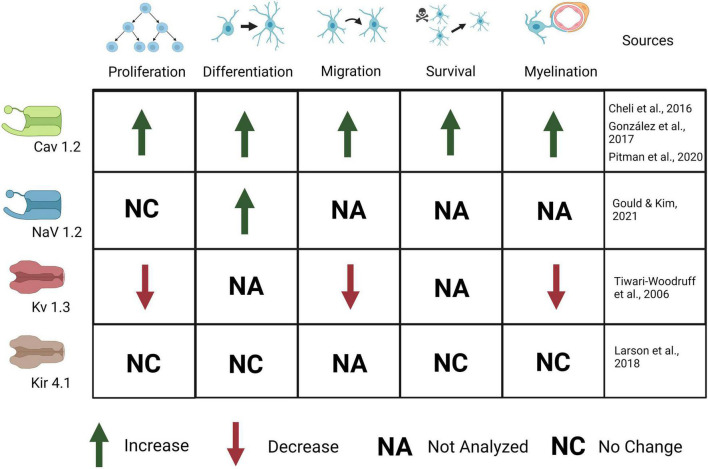
Ion channels expressed in OPCs are important for OPC development and myelination. Activation of CaV1.2 causes an increase in OPC proliferation, differentiation, migration, survival, and myelination. Activation of Nav1.2 causes an increase in OPC differentiation but no change in proliferation. Activation of Kv1.3 causes a decrease in OPC proliferation, migration, and myelination. Kir4.1 activation causes no change in OPC proliferation, differentiation, survival, or myelination, although behavioral and electrophysiological defects (not depicted here) are present.

VGC expression is highest early in postnatal development during synapse formation and myelination, then declines in mature OLs after developmental myelination in adulthood (Kukley et al., 2010; Spitzer et al., 2019). The activation of VGCs is implicated in OPC differentiation and proliferation, and, again, Cav1.2 has proven to be the primary contributor. Knockdown of Cav1.2 increases the presence of OPC markers such as NG2 and Pdgfrα and decreases the presence of OL markers such as CC1 and MBP, indicating maintenance of the progenitor state (Figure 4; Cheli et al., 2016). OPCs in Cav1.2 knockout mice also show decreased proliferation and maturation: OPC processes are much less complex, do not form as many synapses with neurons, and the neurons are less myelinated (Cheli et al., 2015). The migration behavior of the OPCs is also altered, and the velocity of migration and overall distance traveled are both decreased (Cheli et al., 2015). It is critical for OPCs to migrate to their proper destinations following local proliferation to ensure myelination can proceed properly after OPC differentiation and neuron maturation.

The regulation of Ca2+ concentrations in OPCs and their role in healthy and diseased OPC function has been reviewed elsewhere (Paez and Lyons, 2020), so here we will highlight the most recent relevant findings related to Ca2+ signaling in regulating OPC development and myelination. Ca2+ effects in neurons and glia can be mediated by several different Ca2+ sensing proteins, including calmodulin, Ca2+/ calmodulin dependent protein kinases (CAMKs), s100B, and calcineurin. In OPCs so far, CAMKII has been implicated in regulating OPC morphology, process extension, and regulation of myelin thickness (Waggener et al., 2013; Cheli et al., 2019). S100B is expressed in OPCs (Hachem et al., 2007), and its nuclear expression in OPCs is correlated with pre-myelinating OPCs; its expression is downregulated in myelinating OLs (Deloulme et al., 2004); and elevated S100B expression leads to a reduction in OPCs transitioning to mature OLs (Santos et al., 2018). Calcium transients are directly involved in myelin sheath formation during development: the frequency of calcium transients is associated with myelin sheath elongation when high frequency stimuli elicit calcium release; conversely, myelin sheaths are shorter when exposed to lower frequency stimuli and decreased Ca2+ release (Krasnow et al., 2018). Further, high amplitude, long duration Ca2+ transients lead to myelin sheath retractions, and conversely, transient high frequency Ca2+ activity coincides with myelin sheath elongation, consistent with the idea that Ca2+ is instructive for myelin formation and myelin remodeling (Baraban et al., 2018). Depending upon the OPC developmental stage, calcium fluctuations in the soma and in the processes are different, declining in OPC processes and becoming even smaller in the soma throughout development (Krasnow et al., 2018). A recent interesting study has teased this out on a single cell level, combining Ca2+ imaging with single cell RNA-Seq and analyses of proliferation and differentiation in a zebrafish model where cell tracking at this granularity is possible (Hoche et al., 2020). Investigators found that some calcium transients spread from processes to the cell body and were synchronized in neighboring OPCs, consistent with a small local network of connected OPCs. The location of the OPC soma mattered: OPCs that resided in axon-rich areas, vs. neuronal soma-rich areas in the spinal cord had a higher probability of exhibiting Ca2+ transients (Hoche et al., 2020), indicating that there is heterogeneity and local differences in Ca2+ mediated signaling that may not be captured when sampling larger brain regions or circuits. An exciting new study sheds light on the role of these Ca2+ transients in myelin sheath regulation: they used *in vivo* one-photon Ca2+ imaging and found that the Ca2+ transients at myelin domains were independent of neuronal activity, and instead were due to mitochondrial release of Ca2+ in axonal and paranodal myelin itself, suggesting that myelin remodeling is independent of neuronal activity (Battefeld et al., 2019). The implications for therapeutic interventions involving cell autonomous mitochondrial release from OLs are intriguing and warrant further exploration.

### Roles of VGKCs and VGNCs in Oligodendrocyte Precursor Cell Development and Myelination

Depolarization and Ca^2+^ currents in OPCs are modulated by voltage-gated potassium and sodium channels (VGKCs and VGNCs). Previous work has shown that Kv1.3 or Kv1.4 (but not Kv1.5 or kv1.6) channel overexpression results in increased OPC proliferation *in vitro* but has no effect on OPC differentiation (Vautier et al., 2004). In addition, knock down or antibody block of Kv1.3 decreases K+ conductance and results in inhibition of OPC proliferation and migration as well as thinner and disrupted myelin (Figure 4). Further, Kv1.3 is associated with OSP-claudin11, two proteins important for tight junction formation and implicated in autoimmune demyelinating disease, suggesting a role for K+ conductance in both OPC development and myelination (Tiwari-Woodruff et al., 2006). Kv1.3 expression is likely regulated by the cytokine IL-17, as IL-17 application leads to increased Kv1.3 expression and activation, as well as a decrease in OPC proliferation (Liu et al., 2021). This suggests that regulation of VGKCs is important for proliferation as well as Ca2+ mediated effects of plasticity; its role in demyelination after injury will be discussed later. Upon depolarization, OPCs show outward K^+^ currents, mediated by VGKCs (Cheli et al., 2016; Nagy et al., 2017). Outward K+ currents affect the magnitude of depolarization and Ca^2+^ influx in OPCs. The A-type K^+^ channel (which includes Kv1.4) in particular, is important for minimizing postsynaptic potentials in OPCs as it is a potent inhibitor of low-voltage activated Ca2+ channels and therefore regulates Ca2+ concentration, especially in thin processes. Blocking A-type channels significantly increases the amplitude, half-width, and duration of EPSPs in Sun et al. (2016). Less frequent, desynchronized stimuli rarely trigger Ca^2+^ influx in OPCs until A-type channels are blocked, in which case Ca^2+^ influx increases, as does EPSP amplitude (Sun et al., 2016). With frequent stimuli, EPSPs in untreated OPCs resemble those in OPCs treated with A-type K^+^ channel blockers, suggesting that in physiological conditions high frequency stimuli can lead to A-type channel closure and a concomitant increase in Ca2+ influx (Sun et al., 2016). This increased inactivation of A-type channels with frequent stimuli demonstrates the role of A-type K^+^ channels in modulating OPC responses to different levels of input. Because of this, VGKCs may have a critical role in activity-dependent myelination and plasticity.

The combined effects of the VGNCs and VGKCs modulate synaptic responses in OPCs, but less is known about the specific roles of VGNCs in OPCs. VGNCs have also been shown to contribute to EPSPs in OPCs: blocking VGKCs results in increased EPSP amplitude, whereas blocking VGNCs reduces EPSP amplitude in OPCs (Sun et al., 2016). Na+ channel currents are first present in OPCs at E18, peak from P6-P16, and are present though decreased at 9 months of age, the last time point sampled. Na+ channel density is highest from P6 to 16, but Na+ channels are maintained throughout life (Figure 3; Spitzer et al., 2019). OPC proliferation declines after P35, and this decrease correlates with the decline in VGNC density (but not KA/AMPAR or NMDAR density). This differential expression may be important for developmental myelination. VGNCs have been observed in OPCs in development and adulthood, and in both gray and white matter; however, VGNCs are lost upon OPC differentiation, along with synaptic connections to glutamatergic synapses (De Biase et al., 2010; Kukley et al., 2010; Spitzer et al., 2019). A recent paper provides strong evidence for the significance of these channels in OPC function: the authors deleted Nav1.2 channels in OPCs in the cerebellum and brainstem (Figure 4; Gould and Kim, 2021). They found that a subset of OPCs expressed this channel, and that the presence of this channel was essential for spiking properties in OPCs (Gould and Kim, 2021). Deletion of Nav1.2 channels impaired OPC differentiation but did not impact proliferation, indicating that receiving neuronal synaptic inputs in these OPCs may cue them to differentiate into myelinating OLs. Nav1.2 is not the only NaV expressed in the OPC lineage, in addition, NaV1.1, NaV1.3, and NaV1.6 are also expressed (Marques et al., 2016), and the function of these channels in OPCs has not been explored to date. The role of sodium channel function in myelination has not been explored, nor has it been determined if it is important for remyelination after injury.

## Direct Synaptic Transmission

### Synaptic Adhesion Proteins

Synaptic adhesion proteins are expressed in OPCs, and several studies have begun to examine the significance of these proteins on synapse function and myelination (Figure 1). One recent study found that presynaptic vesicle machinery accumulates on axons under myelin sheaths, and that PSD-95 is expressed in myelinating oligodendrocytes at multiple locations on the myelin sheath (Hughes and Appel, 2019). Subsequent RNA-Seq analysis in OPCs found expression of several other synaptic adhesion proteins, including Lrrtm1 and Lrrtm2, Neuroligin 1, Neuroligin 2, Cadm1a, and Cadm1b. Investigators then generated dominant negative alleles for these synaptic adhesion proteins, and found that dnCadm1b, dnLrrtm1, and dnLrrtm2 led to decreased sheath lengths, while dnCadm1b, dnLrrtm1, and dnNlgn2 led to decreased number of sheaths per OL. Cadm1b localized to the myelin sheath membrane, and using dominant negative knockdown of Cadm1b, investigators found that Cadm1b was important for myelin sheath growth (Hughes and Appel, 2019). This work provides strong evidence that synaptic contacts from neurons at the myelin sheath are important for myelin sheath maintenance, but illustrates that much is unknown about the synaptic adhesion proteins and their function in OPCs.

### Oligodendrocyte Precursor Cell Activity During Development

Oligodendrocyte precursor cell responses to glutamate vary depending on the phase of oligodendrocyte lineage development. OPCs show no response to depolarization in the first wave of OPC proliferation in embryonic development in the forebrain, with responses not observed in OPCs until P5 (Ziskin et al., 2007), although there are responses to depolarization in the spinal cord, see Osterstock et al. (2018). However, exposure to glutamate induces mEPSCs in OPCs both in very early postnatal development and into maturity in the forebrain and spinal cord, (De Biase et al., 2010; Spitzer et al., 2019; Tsata et al., 2019), and in both gray (Bergles and Jahr, 2000; Mangin et al., 2008), and white matter (Kukley et al., 2007; Ziskin et al., 2007; Káradóttir et al., 2008; Etxeberria et al., 2010). The degree of response to glutamate differs greatly between OPCs, pre-OLs, and OLs. Where OPCs exhibit mEPSCs in response to glutamate release, pre-OLs respond very infrequently, and OLs do not show any discernable response (Kukley et al., 2010). This could be due to a plasticity-like response as voltage-gated channels are lost along with synaptic connections as OPCs mature to OLs (De Biase et al., 2010). Similarly, the response could also be affected by the decreased surface expression of AMPA and NMDA in OLs (De Biase et al., 2010).

A recent study examined glutamatergic, cholinergic, and GABAergic synapses onto OPCs in the embryonic spinal cord (Osterstock et al., 2018). They found that epithelial cells differentiating into OPCs received axoglial synapses at the same time as the first synapses formed on motor neurons. Furthermore, after migrating to the marginal zone, these OPCs received both functional glutamatergic and GABAergic synapses, and cholinergic signaling potentiated both glutamatergic and GABAergic currents (Osterstock et al., 2018). These experiments suggested that synapse formation triggers differentiation of progenitor cells into OPCs, although whether these synapses are instructive for OPC differentiation and subsequent myelination was not tested.

### Synaptic Activity and Myelination

It has been shown by work from several labs that neuronal activity can increase myelination; this is reviewed elsewhere (Maas and Angulo, 2021). Briefly, increasing neuronal activity using chemogenetics leads to increase in OPC proliferation and differentiation of OPCs, as well as increasing the probability of an axon being myelinated, and resulting in thicker myelin on stimulated axons (Gibson et al., 2014; Mitew et al., 2018). The authors also found that decreasing neuronal activity could decrease the probability of myelination, indicating that myelination is activity-dependent and that neuronal activity is instructive for myelination. Another group found that inhibition of vesicular release, or silencing of neuronal activity, can also have an effect on myelination (Korrell et al., 2019). Inhibition of vesicular release via an OPC-specific knockout of the SNARE protein SNAP-25 led to an overall decrease in MBP protein as well as the amount of myelinated projections in the cortex, and also showed that the myelin node of Ranvier length is also affected (Korrell et al., 2019). Conversely, overexpression of the potassium channel Kir2.1 via in utero electroporation at E13.5 resulted in no change in MBP levels but a significant decrease in node of Ranvier length (Korrell et al., 2019). These studies suggest a mechanism in which neuronal synaptic inputs onto OPCs are instructive for OPCs to differentiate and myelinate. However, these results, while fascinating, do not indicate whether vesicle fusion is necessary for targeting of myelin to a specific location on the axon, for myelin formation, or for growth of the axon sheath.

Other recent work *in vivo* has further resolved the mechanism of neuronal activity on myelin formation, further elucidating the role that presynaptic vesicle release and neuronal synaptic inputs have on myelination. The Lyons lab showed that blocking synaptic release with TeNT decreased myelin sheath length, and increasing synaptic vesicle release with PTZ increased myelin sheath length *in vivo* in a zebrafish model of myelination (Mensch et al., 2015). A subsequent study also from the Lyons lab further resolved the mechanism of this process, imaging individual neurons *in vivo* in zebrafish, and found very interesting results: vesicle fusion increased with myelination, and was localized to hotspots on the heminode, the non-myelinated domain into which myelin sheaths grow (Almeida and Lyons, 2017; Almeida et al., 2021). This suggests that the vesicle fusion is instructive for myelination, and that the presence of myelin also serves as a positive feedback signal for more vesicle fusion and myelin sheath growth.

All of these changes in glutamatergic synapses, from the increased intensity of EPSCs with repetitive stimulation to the changes in receptor expression, resemble long term potentiation and support current theories on activity-dependent myelination. OPC plasticity likely has a significant impact on myelination in development and adaptive myelination in the adult brain. One recent study tested one aspect of plasticity, examining neuronal remodeling during myelination in the second postnatal week in mice (Wang et al., 2021). They found that during axon remodeling, axons that were in competition both received myelin, but the nodes of branches that were maintained had more mature nodes. Myelination itself did not influence competition, and axon branches that were maintained were equally likely to be already myelinated (Wang et al., 2021). This suggests that LTP may contribute to myelin remodeling, although whether these mechanisms are different in developmental myelination compared to adaptive myelination remains to be explored.

## Disease and Treatment

Given the complexity of demyelinating diseases such as MS and the heterogeneity among patients, there is no comprehensive animal model of this disease. Researchers have focused on different animal models to investigate remyelination and understand the mechanisms involved in demyelination in the search of efficient treatments to revert and restore proper circuit function. Many other reviews propose possible new therapies and advances on research related to remyelination (Hooijmans et al., 2019; Kremer et al., 2019; Franklin et al., 2021). In this review we focused on the newest research that showed involvement of ion channels, neurotransmitter receptors, and an explanation of synaptic mechanisms relevant to the OPC-neuron synapse in the context of OPC development and myelination. In this section we discuss the correlation of each channel to a specific neurological disease related to OPC development, myelination, or demyelination, and provide connections to the molecular functions of neuron to OPC synapses to inform our understanding of the mechanisms that underlie demyelinating diseases as well as novel approaches for treatment.

### AMPAR Participation in Injury and Remyelination Involves Calcium Influx

AMPA receptors are the primary mediators of glutamatergic currents in mature OLs, and overactivation can lead to excitotoxicity in OLs. Glutamate mediated Ca2+ influx in OLs is mediated by the GluA4 subunit, and deletion of this subunit decreases Ca2+ responses (Evonuk et al., 2020). An experimental autoimmune encephalitis (EAE) injury model resulted in a significant decrease in myelinated axons. This effect was partially rescued in OL-specific GluA4 knockout mice (Evonuk et al., 2020), indicating that the loss of GluA4 in mature oligodendrocytes, leading to a functional reduction in AMPAR activity, was protective against excitotoxicity damage mediated by demyelinating injury. This suggests that methods that either genetically or pharmacologically block GluA4 function would be a possible therapeutic target in patients with MS.

AMPA is also thought to be the primary receptor involved in remyelination. In studies of demyelinating lesions, AMPA was shown to mediate glutamatergic synaptic activity, recruiting OPCs to the site of injury (Gautier et al., 2015). Synaptic activity directs these OPCs to differentiate and create new OLs to remyelinate the injured areas. Blocking AMPA receptors halts this differentiation and subsequent remyelination. It is important to note that variable Ca^2+^ influx at these sites indicates the presence of both Ca^2+^ permeable and Ca^2+^ impermeable AMPA receptors (Gautier et al., 2015). This effect cannot be directly attributed to calcium influx, but it is consistent with results from synaptic transmission and neuronal activity that require Ca2+ influx. More recently a more specific approach overexpressing subunit GluA2, rendering AMPA receptors Ca2+ impermeable, specifically increased OPC proliferation in the adult brain (Khawaja et al., 2021). After demyelinating injury, OPC proliferation and OL regeneration were also increased after GluA2 overexpression, indicating that Ca2+ levels are critical for post-injury OL formation (Khawaja et al., 2021). These findings suggest that regulating AMPAR signaling is a mechanism to initiate remyelination after brain injury or in chronic demyelinating diseases such as MS. However, precise regulation of Ca2+ concentrations are critical, as elevated levels of Ca2+ may have the opposite effect, and regulation of Ca2+ activity is also necessary to maintain cell health and induce recovery.

The frequency of synaptic events is reduced after demyelination using the LPC model, an endogenous lysophospholipid that disrupts myelin lipids, indicating that OPCs are weakly or not connected in this state, despite the increase in their proliferation (Sahel et al., 2015). In slices kept in culture for one week, spontaneous activity was present and could be blocked by AMPA/kainate receptor antagonists onto reactivated OPCs. The presence of synaptic vesicles was confirmed with 3D confocal reconstruction and electron microscopy, and EdU labeling confirmed the absence of VGlut1 puncta in proliferating cells. Although synaptic activity was present, the results suggested a down-regulation of synaptic inputs in actively proliferating OPCs in demyelinated lesions. But even though the number of connected cells increased after 7 days, the frequency of synaptic currents did not return to control levels. Na+ currents and K+ conductance showed no change after LPC-induced lesions. This indicates that after a demyelination event, major alterations occur in the synaptic connectivity between OPCs and neurons, leading to virtually no synapses during the proliferation phase. Altogether, this might be a beneficial effect in which the decrease of glutamatergic synapses impairs glutamate-dependent calcium signals and facilitates OPC proliferation, which is followed by a subsequent recovery in connectivity between OPCs and neurons in the remyelination phase; this possibility remains to be tested.

Another model that brought insight for OPC synapse function came from nascent synapses onto glioma, which indicates that neuron to OPC synapses can contribute to pathological conditions as well. Researchers found AMPAR-mediated EPSCs as well as potassium currents, and depolarization of glioma contributed to cancer progression and increased neuronal excitability *in vitro*, potentially contributing to neurological deficits in patients with gliomas (Johnson-Venkatesh et al., 2015). This reciprocal activation of neurons activating and enhancing glioma progression, and gliomas promoting neuronal excitability indicates that targeting this synapse could provide a useful therapy for targeting this fatal cancer.

### NMDA Activation Is Important for Remyelination

NMDA could be most relevant in neuron to OPC synapses at later stages. For example, NMDA begins to be expressed in the second week after demyelinating lesions, indicating its importance for later stages of differentiation and myelination (Gautier et al., 2015). Further, D-aspartate, an amino acid that can affect NMDA mediated plasticity, was recently shown to increase OPC differentiation and increase expression of myelin during myelination and remyelination, an effect which was reversed when NMDA antagonists were used (Rosa et al., 2019). A large body of evidence has also shown a role for NMDA in oligodendrocyte mediated excitotoxicity, as NMDAs are expressed both on mature OLs and at the myelin sheath, leading to oxidative stress and damage both to OL cell bodies and myelin sheaths in hypoxia, TBI, MS, and other white matter injuries (Micu et al., 2006; Christensen et al., 2016); reviewed in Matute (2006). Interestingly, AMPA receptors mediate Ca2+ excitotoxicity in OLs, but NMDA receptors mediate Ca2+ excitotoxicity in myelin (Micu et al., 2018). Myelin morphology changes as a result of NMDA but not AMPA receptor activation, with myelin sheath invaginations and disorganized myelin (Christensen et al., 2016). Consistent with this, work *in vitro* has shown that there is an activity- independent pathway, as well as an NMDA-dependent glutamatergic signaling that is critical for myelination, with neuregulin mediating this switch (Lundgaard et al., 2013). Furthermore, the authors found that remyelination after white matter damage was NMDA receptor dependent: pharmacological blockade of NMDA after a demyelinating lesion resulted in a significant decrease in the number of axons that were myelinated as well as myelin thickness after injury. These results indicate that blocking NMDA receptor signaling may be a valid therapy for white matter damage.

### Participation of Voltage-Gated Channels in Disease Models

Additional work has shown that OPC-specific loss of CaV1.2 leads to defects in remyelination after cuprizone treatment (systemic administration of a toxin that targets oligodendrocytes and is a model for MS), to induce demyelination: both OPC and OL numbers were decreased, myelin proteins synthesis and expression levels were reduced, and myelin thickness was reduced in Cav1.2 mutants compared to controls (González et al., 2017). This suggests that the survival signals that OPCs and OLs normally use to prevent cell death after cuprizone-induced lesions are dependent upon CaV1.2 dependent Ca2+ currents and downstream signaling to promote survival. Finally, another group examining a CaV1.2 OPC-specific knockout found that OPC survival was decreased specifically in the corpus callosum but not in the cortex, suggesting that Ca2+-mediated effects are different in gray vs. white matter (Pitman et al., 2020). Together these studies indicate that Ca2+signaling may serve as an important inductive signal for OPC survival and myelination, however, the downstream signal transduction cascades that are responsible for this process have not been determined. It is not known whether this loss is merely the byproduct of synapse loss or if it has any causal relationship.

The voltage gated channel Cav1.2 is also present in astrocytes (Parri and Crunelli, 2003). The conditional knockout of this channel specifically in astrocytes in a cuprizone model of myelin injury resulted in lower microglia and astrocyte activation (Zamora et al., 2020). In addition, general proinflammatory factors such as TNFα and IL1ß were reduced as shown on RT-PCR and ELISA analysis. In this mild inflammatory environment, remyelination was enhanced in Cav1.2 knockout animals when compared to a control group, as shown by thicker myelin sheaths and increased OPC proliferation in the corpus callosum at 2 and 4 weeks of recovery (Zamora et al., 2020). These data suggest that a significant reduction in inflammation is helpful in order for myelin recovery and that inflammation is dependent on the presence of Cav1.2 channels in astrocytes.

Potassium voltage-gated channel Kv1.3, the most prevalent Kv+ channel in OPCs and oligodendrocytes, has increased expression in the inflammation model of myelin injury using IL-17 (Liu et al., 2021). Researchers showed that blocking this channel prevented cell loss and inhibition of OPC proliferation in a demyelinating injury model, and that this effect is mediated by diminishing AKT signaling pathways. This provides evidence for the involvement of KV1.3 in OPC proliferation and differentiation in inflammatory models, suggesting a potential target to prevent myelin damage when inflammatory response is activated in injury and disease.

Another potassium channel broadly expressed in glia, Kir4.1, seems to be involved in myelination and K+ clearance from white matter and its deficits lead to seizures and myelin vacuolization, but the role in oligodendrocytes is not yet clear. OPCs lacking Kir4.1 were more depolarized and exhibited a 10-fold higher membrane resistance than controls, suggesting kir4.1 channels are important for resting conductance of OPCs leading to a hyperpolarized membrane potential. Mice also had a lower threshold for seizures, due to enhanced neuronal excitability, and activity-dependent motor behavior (Larson et al., 2016). This channel seems to have a relevant role in oligodendrocytes to control action potentials and electrophysiological dynamics, but more critical elements such as survival and general myelination are not affected. It is likely that K+ compensating mechanisms take place to prioritize critical functions, but fail to maintain proper K+ dynamics.

### GABA and Remyelination (MS)

The myelination pattern in the uppermost cortical layers is highly variable for most populations of neurons, and myelin regeneration alters the pattern of myelination in cortical circuits (Orthmann-Murphy et al., 2020). The strongest observed pattern is on GABAergic neurons, more specifically PV and somatostatin (SOM) expressing interneurons. PV axons are the most highly myelinated and they exhibit consistent axon density across the primary sensory and motor cortices (Call and Bergles, 2021). After cuprizone treatment, researchers observed a high preference for myelination on the axons of PV neurons, restoring overall myelin content after demyelination, even when the total pattern of myelination along individual axons changed overall. Results also showed that PV axons are selected for remyelination even in regions with lower oligodendrocyte regeneration. The proportion of these neurons on layer I is very low (10%), and doesn’t represent a massive recovery of global remyelination, but this indicates there is some preference for selecting this type of neuron for remyelination.

Interestingly, lesions of demyelination resulting from MS are reduced in pregnant women due to enhanced myelination (Kalakh and Mouihate, 2019). Investigating this effect further, a study showed that the manifestations of remyelination were linked to GABA receptor activation. In this study, the group that received bicuculline (a GABAR antagonist) showed fewer remyelination indicators such as reduced density of NF+ fibers, reduced density of MBP+ fibers and a decreased myelination index, as well as reduced density of OPCs in the demyelinated area, followed by a reduction in the expression of MAG, a myelin protein with a periaxonal localization, in the corpus callosum when compared to the control pregnant demyelinated group. The increased GABAergic tone during pregnancy is naturally mediated by the progesterone metabolite ALLO7. The affinity of ALLO7 to GABAR is higher with the presence of the γ2 subunit, which is one of the isoforms that has significantly higher expression in pregnant animals, and might mediate the increase in OPC proliferation associated with the activation of GABA_*A*_R. Therefore, this study shows a mechanism by which MS pregnant patients present reduced symptoms associated with demyelination, and may bring insights for future treatments.

### Therapies

New therapies to enhance remyelination offer directions for new treatments for patients with demyelinating injury, including hypoxia, white matter injury, multiple sclerosis, TBI, and also developmental disorders such as autism and schizophrenia. Recent experiments have shown that ion channel inhibitors can rescue some of the defects found in the demyelinating model of cuprizone treatment (Gopalasingam et al., 2019) and partial optic nerve transection (O’Hare Doig et al., 2017). Specifically, a cocktail of ion channel inhibitors to block calcium entry partially rescued OPC loss and decreased the number of atypical nodes of Ranvier, although paranode length decreases were not rescued, indicating that excessive calcium is an important contributor of demyelinating injury. The downstream mechanisms of calcium signaling that lead to OPC loss and regulate node of Ranvier size have yet to be determined.

Recent studies found that chronic hypoxia led to a decrease in oligodendrocytes and hypomyelination (Wang et al., 2018). Defects in synapse formation, as measured by quantification of synaptic puncta, and synapse function, as measured by mEPSC recordings, were also present. Knock out of M1R, a muscarinic receptor that negatively regulates OL differentiation in OPCs, rescued the defects in OPC survival, as well as the hypomyelination and synaptic defects. Clemastine, a M1R antagonist, was also effective at rescuing hypomyelination and restoring motor and cognitive behavioral deficits (Wang et al., 2018). This suggests that regulating OPC survival is not only protective for recovery from myelin injury, but is beneficial for rescuing synaptic function in hypoxia. Clemastine has also showed effects on remyelination in other disease models such as compression neuropathy (Lee et al., 2021), Alzheimer’s disease (Xie et al., 2021) and MS (Green et al., 2017). The mechanisms of action are still unclear, but tests showed prevention of OPC loss, an increase in myelination expression, prevention of senescence (Xie et al., 2021), an increase in myelin thickness, and a decrease in the latency of impulse conduction (Lee et al., 2021). Clemastine fumarate is a first generation H1 anti-histamine used to relieve allergic reactions by competing with H1 receptors, other than M1R. As a drug already in use it shows promising application for remyelination treatments.

While many therapies focus on promoting oligodendrogenesis and differentiation to promote remyelination, a recent study (Duncan et al., 2018) showed existing adult oligodendrocytes are capable of generating new myelin sheaths. Although the authors did not confirm that newly generated oligodendrocytes were not participating and responsible for the remyelination, the pattern observed in 2D and 3D light and EM microscopy lead to the conclusion that mature oligodendrocytes are connected to mature and remyelinated myelin sheaths (Duncan et al., 2018). Remyelination by adult oligodendrocytes may be more limited especially in the middle of MS lesions, but OLs may extend processes in the plaque periphery. Considering one of the models used (vitamin B12-deficiency), B12 therapy may improve the restoration and function of preexisting oligodendrocytes, the participation of mature oligodendrocytes in remyelination may play complementary roles in myelin repair in the CNS and open new therapeutic approaches to improve patients’ recovery.

A greater understanding of OPC regeneration could come from examining the electrophysiological properties of OPCs in animals that are able to undergo axonal regeneration and remyelination. For example, adult zebrafish, unlike mammals, are capable of regenerating OLs and remyelinate axons within 2 weeks of lesion. This indicates that zebrafish glia could be a useful model for mammalian remyelination after injury or disease (Tsata et al., 2019). The basic channel properties and expression in zebrafish OPCs were similar to mammalian OPCS, which opens up the possibility that determining how OPCs differently respond in this model could aid our understanding of remyelination in mammals.

## Discussion

Synaptic activity between neurons and OPCs has been shown to affect OPC proliferation, migration, maturation, and differentiation. In this review we discussed the most recent findings involving synapse function and receptors expression in different phases of cell development, and their impact in disease models that are relevant to direct possible therapeutic research and approaches. However, more research is needed to better understand these mechanisms since many gaps are still present. In early development, the central nervous system undergoes significant pruning of unnecessary synapses, and synaptic plasticity continues to change connectivity in the adult nervous system. Repetitive neurotransmitter release and sustained neuronal firing causes increased Ca2+ entry via NMDA receptors, activating CAMK and calmodulin mediated immediate early gene transcription and downstream signal transduction cascades that lead to insertion of new AMPA receptors and new AMPA protein synthesis, as well as dendritic spine growth, increasing the ability of the postsynaptic cell to respond to neurotransmitter release (Malenka and Bear, 2004), resulting in LTP at the synapse. Long term depression conversely causes spine shrinkage. LTP has also been observed in OPCs, but the significance of NMDA signaling in OPCs in developmental myelination is inconclusive, with contradictory research showing no change in OPC proliferation, morphology and differentiation on one side and a critical role in migration, synapse formation, OPC differentiation and myelination on the other. It may be that the relationship of NMDA to AMPA in glutamatergic signaling, while still unclear, can represent compensatory mechanisms at play, with AMPA as the main receptor regulating OPC activity and NMDA supporting and fine-tuning it, especially in mature OLs where NMDARs are more present. Additional experiments will be necessary to determine this interplay.

The roles of VGNCs are also poorly understood, which is particularly important given that they are responsible for OPC depolarization but their diversity makes the task very challenging. Still, we discussed how calcium transients are involved in myelin formation and regulation and considering that appropriate calcium levels play a critical role in regulating cell survival especially in injury models, and Ca2+ is a critical signal for subsequent remyelination, it is of high importance to better understand the regulation of calcium in OPCs, which may have considerable implications for therapeutic interventions.

Last, the function of GABAergic synapses in OPCs is still unclear. At a glance, OPC depolarization does not have a marked effect but is still relevant on changing intracellular Ca2+ concentration. More pertinent is the role of GABA on OPC survival and myelination. It is still an open question how GABAergic activity acts upon proliferation, as inhibition of neuronal activity via GABAergic neuronal activity does not have the same effects as the direct action of GABA on OPCS. Better knowledge of these mechanisms will increase our understanding of the role of neuron to OPC synapses in myelination.

The implications of synaptic activity for OPC differentiation and myelination are analogous to the mechanisms of synaptic plasticity (Reviewed by O’Rourke et al., 2014, Mount and Monje, 2017). The relationship with neurons is bilaterally relevant because it was observed that LTP in neurons was enhanced after oligodendrocyte depolarization and reduced the magnitude after oligodendrocyte hyperpolarization (Ge et al., 2006). The activation of NMDA receptors and voltage-dependent channels facilitates the coordination of axonal induction through additional depolarization and enhanced LTP induction. The opposite effect causes oligodendrocyte hyperpolarization, inhibiting the activation of NMDA receptors and subsequently suppressing LTP induction. These results suggest a more important participation of oligodendrocytes in cognitive processes, learning, and memory. Further examination of the role of LTP and plasticity in myelination is an important future step to unraveling the mechanisms of myelination. If LTP-like processes impact OPC differentiation and myelination, activity could continue to influence OPC differentiation in the adult CNS.

Activity-dependent, or adaptive, myelination is also increased by increased neuronal activity, driven by factors such as motor learning and sensory experience. For example, myelination is increased by social interaction. Isolation early in development causes decreased myelination, and is only rescued if social reintroduction occurs early enough (Makinodan et al., 2012). In order to better understand the relationship between synaptic activity and myelination processes, more research is also needed on the downstream effects of synaptic activity. For example, brain-derived neurotrophic factor (BDNF) has been shown to regulate myelination (Vondran et al., 2011). Oligodendrocyte-derived BDNF functions via presynaptic tropomyosin receptor kinase B (TrkB) and the reduction of BDNF in mutant mice decreases vesicular glutamate release by reducing the available pool of glutamate vesicles. This reduction in endogenous BDNF does not alter the Ca2+ channel activation or Ca2+ influx in presynaptic terminals. In mutant mice, the amplitude of EPSCs is significantly smaller in immature and mature synapses (Jang et al., 2019), and mice exhibit decreased numbers of myelinated axons, and decreased PLP and MBP mRNA (Vondran et al., 2011).

Recent advances in imaging and genetics have allowed us to tackle some of the most challenging questions around the development of neuronal circuits and their role in myelination. One group has used semliki forest virus vectors to create 3D reconstructions of OL processes and internodes (Toth et al., 2021). Using this technique is it possible to analyze how local neuronal activity in a brain slice can change internode length and process branching, thus tackling the challenging task of understanding the dynamics of myelin changes in response to neuronal activity. The Bergles lab has created a new tool for *in vivo* tracking of oligodendrocytes, Olig-Track, which can track thousands of OPCs, conduct volumetric segmentation, and analyze soma size over weeks *in vivo* (Xu et al., 2021). This method relies on cranial windowing, *in vitro* two photon microscopy, and a deep learning approach based on segmentation, training, seed extraction, and cell tracking to reliably and robustly track cells over time *in vivo*. This method is significantly more accurate than a heuristic model and provides a powerful new approach to examine oligodendrocyte development, migration, and maturation *in vivo* (Xu et al., 2021). These new methodologies will be valuable to examine the effectiveness of new therapeutics in rescuing oligodendrocyte defects and white matter changes *in vivo*.

Finally, evaluating the efficacy of treatments promoting remyelination is limited and challenging. MRI is the most common measure to assess white matter but it fails to provide reliable and sensitive metrics (Petiet et al., 2019). In the search for new methods to evaluate remyelination, Heidari et al. (2019) quantified visual evoked potentials (VEP) latencies and efficiently correlated with the functional recovery of remyelination along the optic nerve, confirmed also by histological analysis. After inducing demyelination on the Optic Nerve (ON) of cats with the feline irradiated diet-induced demyelination (FIDID) model they tracked the course of disease and associated with the progressive decrease in the VEP latencies. The subsequential functional recovery followed the same protocol, and histological comparison of stages was evaluated quantifying the myelin thickness (g-ratio). Myelin status of the ON was significantly correlated with VEP latency and low remyelination rates; additionally, remyelination was delayed. This method relies on functional and structural integrity of the retina and central visual pathways, but is a promising new approach for measuring functional recovery of myelination *in vivo*.

In conclusion, myelination is a complex mechanism coordinated by cell-cell interactions, and a direct result of OPC proliferation, survival and differentiation dynamics. The work reviewed here demonstrates that highly complex regulatory signaling systems occur to control oligodendrocyte development and CNS myelination. While we have come a long way to understand the mechanisms involved in myelination, demyelination and remyelination, there are still many gaps of information on how these processes occur in detail and how we can efficiently manipulate them to provide adequate diagnosis and treatment for demyelinating diseases.

## Author Contributions

LC determined the scope of the manuscript and figures. LC, DM, and EB co-wrote the manuscript. RB created the figures. All authors contributed to the article and approved the submitted version.

## Conflict of Interest

The authors declare that the research was conducted in the absence of any commercial or financial relationships that could be construed as a potential conflict of interest.

## Publisher’s Note

All claims expressed in this article are solely those of the authors and do not necessarily represent those of their affiliated organizations, or those of the publisher, the editors and the reviewers. Any product that may be evaluated in this article, or claim that may be made by its manufacturer, is not guaranteed or endorsed by the publisher.
